# Radiation safety management for stereotactic body radiation therapy incidentally concurrent with Pluvicto® (Lu‐177) therapy ‐ a case report

**DOI:** 10.1002/acm2.70746

**Published:** 2026-08-16

**Authors:** Dongxu Wang, Bae P. Chu, Maria F. Chan, Jeonghoon Park, Yolanda Scott, Puneeth Iyengar, Matthew Williams

**Affiliations:** ^1^ Department of Medical Physics Memorial Sloan Kettering Cancer Center, Basking Ridge New Jersey USA; ^2^ Department of Medical Physics Memorial Sloan Kettering Cancer Center New York New York USA; ^3^ Department of Radiation Oncology Memorial Sloan Kettering Cancer Center, Basking Ridge New Jersey USA

**Keywords:** external‐beam radiation therapy, radiation safety, radiopharmaceutical

## Abstract

A patient with castration‐resistant prostate cancer (CRPC) was referred to our clinic seeking palliative management of a painful metastasis in the left pelvis. At the time of referral, the patient was receiving [177Lu]Lu‐PSMA 617 (Pluvicto®) therapy at an outside institution, with the next scheduled administration approaching within days. As the patient's next Pluvicto® cycle could coincide with the planned SBRT, the radiation oncologist consulted with the medical health physics team to evaluate potential radiation safety concerns and to determine an appropriate treatment timeline. This case report details the radiation safety considerations, dose estimations, and the measured dose rate readings on the patient to balance the optimal patient care and to minimize staff exposure.

## 1 INTRODUCTION

A 90‐year‐old patient with castration‐resistant prostate cancer (CRPC) was referred to our clinic seeking palliative management of a painful metastasis in the left pelvis. At the time of referral, the patient was receiving [^177^Lu]Lu‐PSMA‐617 (Pluvicto^®^) therapy at an outside institution, with the next scheduled administration approaching within days. Patient reported the improvement of pain in most other sites with systemic therapy, but the pain in his left iliac bone persisted. The radiation oncologist recommended prompt stereotactic body radiation therapy (SBRT) to the left iliac/pelvis disease, the only site of pain that required urgent palliation. As the patient's next Pluvicto^®^ cycle could coincide with the planned SBRT, the radiation oncologist consulted with the medical health physics team to evaluate potential radiation safety concerns and to determine an appropriate treatment timeline. This case report details the radiation safety considerations, dose estimations, and measured dose rate readings on the patient to balance the optimal patient care and to minimize staff exposure.

## 2 TECHNICAL BACKGROUND

Lutetium‐177 (Lu‐177) primarily emits beta particles (E_max_ = 498 keV, 79%) with low‐energy gamma photons, mainly at 113 keV (6.4%) and 208 keV (11%)^1^. It decays with a physical half‐life of 6.6 days, and an *effective* half‐life of 41 hours^2^ with physiological process included. Pluvicto^®^ is typically administered as an intravenous injection of 7400 MBq (200 mCi) once every 6 weeks for up to six doses. In cases of treatment‐related toxicity, the administered activity may be reduced to 5920 MBq (160 mCi)^1^.

## 3 REGULATORY FRAMEWORK

The United States Nuclear Regulatory Commission (NRC) allows the release of patients who have received radioactive material from a hospital's control if it is determined that the radiation exposure to any member of the public is not likely to exceed 5 mSv total effective dose equivalent (Code of Federal Regulations Title 10, Part 35.75, i.e., 10 CFR 35.75)^3^. Guidance for implementing this regulation, including methods for assessing patient‐specific release criteria and estimating public exposure, is provided in NRC Regulatory Guide 8.39: Release of Patients Administered Radioactive Material^4^. Patients who receive Pluvicto® injection are typically releasable from the hospital on the same day with instructions on radiation protection precautions, for example, limiting close contact with others and children/pregnant women for specified durations. While these precautions are designed to protect members of the public, healthcare staff who handle or interact with radioactive patients are subject to a separate set of requirements concerning occupational dose.

The NRC defines occupational dose as the radiation an individual may receive from all licensed and unlicensed sources while performing their assigned duties, regardless of whether the source is under the control of the licensee or another person. **In its definition of occupational dose** ^5^ **, the NRC specifically excludes exposure from patients who have been administered radioactive materials and released** in accordance with 10 CFR 35.75. This exclusion means that such a patient's dose is not separately tallied against the clinic's occupational dose records. In practice any such dose is captured within routine personnel dosimetry and cannot readily be deconvoluted from other occupational exposure. Recognizing that a patient could carry substantial residual radioactivity while undergoing care at our facility, the authors determined that the prudent approach was to implement appropriate radiation safety precautions as necessary. The following sections describe the authors’ procedures and observations in applying these safety measures.

## 4 ESTIMATING RADIATION EXPOSURE RATE FROM ADMINISTERED LU‐177

A literature search revealed that there is limited published experience describing the delivery of radiation therapy to patients who are *concurrently receiving* radiopharmaceutical therapy; no radiation safety studies specifically addressing the scenario described in this case report were identified. While a few studies have investigated combined or sequential administration of radiopharmaceutical therapy with external beam radiotherapy^6^, ^7^, these studies focus on treatment efficacy, dosimetry, or clinical outcomes, rather than the radiation safety implications for staff and facility operations during concurrent treatments. There is a notable gap in the literature regarding practical radiation safety measures. This case study seeks to address the gap by providing an evidence‐based description of the safety protocols, measurements, and considerations for external‐beam radiation therapy patients who retain residual radioactivity from an administered radiopharmaceutical.

Relevant data on external dose rates help contextualize these safety protocols. Bellamy *et al* ^8^ reported that the average external dose rate of Lu‐177 patients was approximately 0.0037 µSv/h per MBq at 1 meter, based on measurements from over 200 patients. Notably, there was substantial variability, with some patients exhibiting dose rates up to twice the average. For radiation safety purposes, applying a factor‐of‐two margin is considered acceptable when estimating exposure on an order of magnitude basis. To better characterize exposure in their own clinical setting, the authors utilized a readily accessible database of patient dose rate measurements at their flagship hospital. Dose rates per MBq were collected from 874 Pluvicto^®^ patients immediately after administration and are summarized in Table 1, providing a foundation for evaluating potential occupational exposure during concurrent radiation therapy.

**TABLE 1:** Average dose rates immediately following Lu‐177 Pluvicto^®^ administration (*N* = 874; February 2019‐May 2025; mean administered activity: 7370 MBq;1 mR is approximately equal to10 µSv in converting exposure to dose equivalent).

Distance from Patient	Dose Rate
At Contact	0.057 (+/− 0.022) µSv/h/MBq
At 30 cm	0.017 (+/− 0.035) µSv/h/MBq
At 1 m	0.0032 (+/− 0.0005) µSv/h/MBq

Based on the average dose rate value reported above, and taking into account the patient's scheduled subsequent cycle of Lu‐177 Pluvicto^®^ administration of 5920 MBq (or 160 mCi), the projected dose rates from the patient were calculated in Table 2.

**TABLE 2:** Estimated dose rates (µSv/h) following Lu‐177 Pluvicto^®^ administration (5920 MBq, or 160 mCi) on Day 0. Dose rates in Day 1 through 7 were calculated by multiplying the dose rate “constant” (µSv/h/MBq) in Table 1 by the residual Lu‐177 activities on each day. Injection activity was obtained from the patient's medical record via the Care Everywhere platform in Epic (Epic Systems, Verona, WI).

Distance from Patient	Day 0	Day 1	Day 2	Day 3	Day 4	Day 5	Day 6	Day 7
Dose Rate at Contact (µSv/h)	336	225	151	101	67.9	45.5	30.5	20.4
Dose Rate at 30 cm (µSv/h)	99.2	66.5	44.6	29.9	20.0	13.4	9.0	6.0
Dose Rate at 1 m (µSv/h)	19.2	12.9	8.6	5.8	3.9	2.6	1.7	1.2
Estimated Residual Lu‐177 Activity (MBq)	5920	3970	2660	1780	1200	800	540	360

## 5 ESTIMATING STAFF EXPOSURE AND DETERMINING SBRT TIMELINE

Although the NRC does not separately count exposure from patients released under 10 CFR 35.75, the authors elected to estimate potential exposures to staff and other patients as a conservative measure. Estimated exposures for specific clinical roles during the patient's care are summarized in Table 3 and were used to guide scheduling of the patient's SBRT while maintaining best practices in radiation safety.

**TABLE 3:** Staff dose equivalent estimates (µSv) based on dose rates from Table 2, accounting for time points, presumed occupancy (roles) and presumed distances. Time and distance for staff roles were estimated based on authors’ clinic's workflow for a typical SBRT patient.

	Time Spent with Patient (min)	Distance from Patient (cm)	Potential Staff Dose Equivalent (µSv)							
			Day 0	Day 1	Day 2	Day 3	Day 4	Day 5	Day 6	Day 7
Radiation therapist	15	30	25	17	11	8	5	3	2	2
Radiation oncologist	45	30	75	50	34	23	15	10	7	5
Nurse	5	30	8	6	4	3	2	1	1	1
Medical physicist	1	30	2	1	1	1	0.3	0.2	0.2	0.1
General staff	60	100	20	13	9	6	4	3	2	1
Other (e.g. other patients sharing waiting room)	60	30	100	67	45	30	20	14	9	6

Upon reviewing the estimated exposures, and accounting for the time required for SBRT treatment planning, the authors determined that the optimal timeline was to perform SBRT simulation 1 to 3 days before Day 0 (defined as the scheduled date of the next cycle of Pluvicto^®^ injection), followed by the delivery of the 3‐fraction SBRT course on Days 7–9. This approach ensures that during SBRT treatment the patient's exposure rate at any distance is expected to remain below 20 µSv/h, or below 2 mR/h (approximately 1 mR = 10 µSv in converting exposure to dose equivalent), consistent with the NRC dose limit of less than 2 mR in any hour for a member of the public. Because this per‐hour value was applied without crediting the actual staff‐patient contact time—typically only minutes per interaction rather than a full hour—the resulting constraint is intentionally conservative and overestimates the dose any single individual would receive. Although radiation therapists are classified as radiation workers with an occupational limit of 50 mSv/year, we selected the 20 µSv/hour threshold to protect *all individuals* in mixed‐use areas where the patient could access, including unmonitored staff and members of the public, as well as to accommodate a declared‐pregnant worker. For this patient, the estimated exposure associated with the therapists’ tasks was low enough that no restrictions beyond routine ALARA practices were warranted. This conservative criterion supported transparent communication and a practical safety plan for our clinic layout.

## 6 RADIATION SAFETY EDUCATION TO STAFF

Following potential exposure calculations to staff, the medical physics team provided targeted education to clinical staff involved in the patient's care. The anticipated exposure to radiation therapists was explained to be within the range typically encountered during standard external beam radiation therapy treatments, and well below regulatory limits. These exposures were also noted to be comparable to, and in fact lower than, those encountered during routine PET/CT simulation procedures, a standard component of external beam radiation therapy planning. Based on institutional measurements, the exposure rate immediately following administration of approximately 160 mCi of Lu‐177 is approximately 10 mR/h at 30 cm; using an effective half‐life of about 40.5 hours, this falls to approximately 0.9 mR/h at six days after administration, corresponding to the timing of the SBRT course. For comparison, Quinn *et al* 9 reported exposure rates of approximately 11 ± 4 mR/h at 30 cm from patients about one hour after administration of 12 mCi of F‐18 FDG for PET/CT simulation^9^. The exposure rate encountered during this patient's radiation therapy was therefore roughly an order of magnitude lower than that routinely encountered during PET/CT simulation, reinforcing that the exposure potential to radiation therapists was low and that time, distance, and shielding measures were sufficient to keep doses well within ALARA goals. Some practical workflow modifications were implemented, such as rotating therapist assignments and establishing an isolated patient walking pathway to minimize incidental contact with other patients and staff. These efforts helped address staff anxiety about the unfamiliar radiopharmaceutical therapy. All staff were informed of radiation safety precautions, including contamination precaution, with specific attention to employees with declared pregnancies, in accordance with best practices.

## 7 RADIOACTIVE CONTAMINATION CONSIDERATIONS

Although the primary concern for external dose is from the residual activity retained inside patient, potential contamination pathways—principally urine—were also considered. Experience at the authors’ main campus with Lu‐177 patients returning for urgent care indicates that contamination from routine contact becomes negligible within approximately three days, and patient hygiene precautions are provided for six days after administration. Because this patient was to receive his SBRT on Day 7 after his prior Pluvicto® cycle and was neither incontinent nor catheterized, residual contamination, including from urine, was not considered a meaningful risk to staff. Standard universal precautions—disposable gloves and hand hygiene—were nonetheless observed. The authors’ outpatient facility is not licensed for Lu‐177 but is licensed for unsealed diagnostic nuclear medicine; on‐site nuclear medicine technologists were available to respond to, survey, and mitigate any urine spill, and any items found to be contaminated would have been held on‐site for radioactive decay. No contaminated items were generated during this patient's care.

## 8 RADIATION SURVEY AT SBRT TREATMENT

During the first fraction of SBRT (Day 7 after Pluvicto^®^ injection), a medical physicist measured the patient's radiation exposure using a calibrated ion chamber survey meter (Fluke 451B, Fluke Biomedical, Everett, WA). These readings were compared with the estimated exposure rates derived from institutional population data, as shown in Table 4. The measured and estimated values were in close agreement, consistent with expectation that individual patient exposure rate may vary by up to a factor of two from the population average.

**TABLE 4:** Measured and Estimated Radiation Exposure Rates from a Patient Administered Lu‐177 Pluvicto ^®^ on Day 7; 1 mR = 10 µSv in converting exposure to dose equivalent.

Distance from patient	Measured dose rate (µSv/h)	Estimated dose rate from population data (µSv/h)^*^
Contact	18	20
30 cm	7	6.0
1 m	2	1.2

* Estimates derived from institutional population data present in Table 2.

## 9 SUMMARY

This case report presents radiation safety considerations for a patient receiving SBRT with a cycle of Pluvicto^®^ (Lu‐177) therapy. Although such precautions were voluntary and not required under current regulatory guidance, the proactive estimation and communication of staff exposure helped ensure safe continuity of care and promote transparency. Literature review and calculations were used to estimate potential radiation exposure to staff and the general public. In our case, the SBRT timeline was established as follows: simulation 1–3 days before Pluvicto^®^ administration and the 3‐fraction treatment on Days 7–9 post‐injection. Radiation survey readings during the first fraction of SBRT (Day 7) were consistent with the estimates within a factor of 2, reflecting the known variability in patient radiation emission. The patient successfully completed treatment without incident. The timeline of SBRT relative to Pluvicto administration may be adjusted in different clinical scenarios and different logistical contexts; this case report presents the methodology and data for making such a balanced decision in other situations.

## AUTHOR CONTRIBUTIONS

All authors participated in the clinical care or radiation safety work of the patient presented in this case. All authors contributed to the writing and editing of the article, and approved the final version.

## CONFLICT OF INTEREST STATEMENT

DW reports receiving licensing fee payment from Ion Beam Applications, S.A. MC reports receiving research funding from Ashland, Inc. Both are outside of this work.

## ETHICS STATEMENT

Data presented in this case were obtained from a clinical patient of the authors’ institution. The patient has signed consent to the use of their deidentified clinical information for teaching purpose only.

## Data Availability

Data presented in this case were obtained from a clinical patient of the authors’ institution. The patient has signed consent to the use of their deidentified clinical information for teaching purpose only. The patient's data will not be shared, other than those presented in this Teaching Case.
